# Age-Specific Breast Density Changes in Taiwanese Women: A Cross-Sectional Study

**DOI:** 10.3390/ijerph17093186

**Published:** 2020-05-04

**Authors:** Yu-San Liao, Jia-Yu Zhang, Yuan-Chi Hsu, Min-Xuan Hong, Li-Wen Lee

**Affiliations:** 1 Department of Diagnostic Radiology, Chang Gung Memorial Hospital, Yunlin 63862, Taiwan; mm601200@gmail.com (Y.-S.L.); lisa20312@gmail.com (J.-Y.Z.); papaya7066@gmail.com (Y.-C.H.); xuan051682@gmail.com (M.-X.H.); 2 Department of Chemistry and Biochemistry, National Chung Cheng University, Chiayi 62102, Taiwan; 3 Department of Nursing, Chang Gung University of Science and Technology, Chiayi 61363, Taiwan; 4 Department of Diagnostic Radiology, Chang Gung Memorial Hospital, Chiayi 61363, Taiwan

**Keywords:** age, mammography, breast composition, mammographic density

## Abstract

Breast density is a risk factor for breast cancer. This study explored distribution of mammographic density quantitatively and qualitatively in a wide age range of Taiwanese women. Subjects with negative and benign mammographic findings were included. According to the Breast Imaging Reporting and Data System, the proportion of extremely dense breasts declined from 58.0% in women < 30 years to 1.9% in women > 74 years. More than 80% of mammograms in women < 55 years old were classified as extremely or heterogeneously dense, while the proportion of dense breasts was still high in women aged 60–64 years (59.3%). The absolute dense area of the breast declined from 35.8% in women < 30 years to 18.5% in women > 74 years. The correlation between breast density and age was significant, with and without controlling for the effect of body composition (*p* < 0.001), implying that the relationship between breast density and age was not wholly related to body composition. In conclusion, the higher breast density in Taiwanese women aged 60–64 years was comparable to that of Western women aged 40–44 years in the literature. This suggests that breast cancer screening using mammography may be more challenging for Asian women than for Western women of the same age.

## 1. Introduction

Screening mammography is a periodic mammogram used to early detect and diagnose breast cancer in women without breast symptoms [1,2]. Mammography is the most effective imaging modality for the detection and diagnosis of breast cancer; however, it may have adverse consequences, such as pain, anxiety, false-positive results, overdiagnosis, overtreatment, radiation exposure, and increased costs [3]. Therefore, the decision about starting age and frequency of screening mammography should be based on a cost-benefit analysis, while taking into consideration the local epidemiology of breast cancer, screening performance, and limitations of mammography.

For breast cancer, the main epidemiological differences between women in Asian and Western countries are the overall lower incidence rates and younger age of peak incidence in Asian women [4,5]. In Western countries, breast cancer incidence peaks at 60–80 years [6,7], whereas in Asian countries, it plateaus or decreases after 45–50 years [4,8,9,10,11]. In Asia, the peak incidence of breast cancer occurs in younger women compared to women in Western countries. However, age stratification study showed similar incidence rates of breast cancer in younger Asian and Western populations [10]. The differences in the incidence of breast cancer between generations in Asian women may due to the westernization of lifestyle in the younger generations and introduction of national screening programs. The diverging incidence trends observed among Asian and Western countries may influence government policies about screening mammography.

There is no consensus regarding the starting age and age ranges for population-based screening mammograms, and recommendations vary across countries [12,13,14]. Nevertheless, screening mammography is generally considered beneficial for women aged 50–69 years [14]. The American College of Radiology, the Society of Breast Imaging, and the American College of Obstetricians and Gynecologists all recommend regular screening mammograms for women starting at 40 years; however, the American Cancer Society recommends starting at 45 years. In Asia, the recommended age to begin population-based screening mammography is 40 years in Japan [15] and Korea [16], 45 years in Taiwan [17], and 50 years in Singapore [18]. In general, regular mammograms are not recommended for women < 40 years of age because of the relatively lower incidence of breast cancer and the higher density of breast tissue.

Breast density is defined as the relative amount of fibroglandular tissue to radiolucent fatty tissue on a mammogram. Women with dense breasts have reduced sensitivity of mammography due to a masking effect, and are at higher risk of developing breast cancer [19]. Therefore, the prevalence of women with dense breast tissue should be carefully considered when establishing a screening mammography program. Currently, age-specific mammography density has been well-established in women > 40 years as some screening protocols begin at 40 years of age. However, little is known about the distribution of breast density, as demonstrated on mammography, among the general population of women < 40 years. Even worse, very little is known of the breast density distribution in women from a wide range of ages using the current Breast Imaging-Reporting and Data System (BI-RADS) 5th edition.

The benefits of extending mammography screening to younger women may be limited and the exposure of healthy young individuals to ionizing radiation through such an inefficient screening tool may be unethical. Alternative studies such as magnetic resonance imaging and ultrasound can be recommended for young women at higher risk for breast cancer. However, some studies have included women aged < 40 years [20,21,22,23,24]. Possible motivations for younger women to engage in early mammography screening may be fear of breast cancer, overestimation of their personal breast cancer risk and lack of awareness of the limitations of screening mammography [22,25]. Given the problems above, scientific evidence is needed to clarify the relative risks and benefits.

In this study, we aimed to determine the age-related breast density in a wide age range of Taiwanese women using qualitative and quantitative methods. An additional aim was to determine the relationship between the two methods.

## 2. Methods

### 2.1. Design, Sample, and Setting

Data for this retrospective analysis are from a community-based, annual health check program at a local hospital (Chang Gung Memorial Hospital Yunlin Branch) funded by a private organization. Participation in this program was voluntary and for free to local residents of Mailiao and Taixi, rural townships in southwestern Taiwan, with a total population of approximately fifty thousand. Women were eligible for screening mammography if they had no breast implants or an indication for a diagnostic mammogram. For women aged > 40 years, routine screening mammograms were recommended every two years. For women aged < 40 years, screening mammography was not recommended except for those with a higher than average risk of breast cancer. A doctor’s referral was not needed but was recommended for young women. However, no precise age-threshold was set for this screening mammography program.

This study was approved by the Chang Gung Medical Foundation Institutional Review Board with a waiver of informed consent (No. 201800154B0 and 201801125B0). Women who underwent screening mammograms between January 2016–June 2018 were reviewed. All imaging reports were interpreted by one radiologist with 14 years of mammography experience, according to the 5th edition of BI-RADS [26]. The final mammographic assessment was classified as follows: category 0, incomplete; category 1, negative; category 2, benign; category 3, probably benign; category 4, suspicious of malignancy; category 5, highly susceptive of malignancy; and category 6, known biopsy-proven malignancy. Subjects with negative (BI-RADS category 1) or benign (BI-RADS category 2) mammographic results were included in the final analysis. For premenopausal women, mammography was scheduled during the first and second weeks of the menstrual cycle. Body composition was measured at the time of mammography or a different time. The duration between mammography and body composition measurements was calculated.

### 2.2. Mammography Density

All enrolled subjects had routine bilateral two-view screening mammograms performed on a full field digital mammography scanner (Senographe Essential; GE Medical Systems, Buc Cedex, France). Breast composition was classified according to the 5th edition of BI-RADS [26] as follows: category A, almost entirely fatty; category B, scattered areas of fibroglandular density; category C: heterogeneously dense; and category D: extremely dense. Categories C and D were combined and categorized as dense, categories A and B were combined and categorized as fatty. In addition to the original reporting, the second reporting of breast composition was performed in a subset of randomly selected mammographic studies (n = 50). The mammograms were read by the same radiologist at least 3 months apart to avoid any recall bias.

The Digital Imaging and Communications in Medicine (DICOM) format of the right craniocaudal mammogram was imported into the Fiji software (https://imagej.net/Fiji) [27] for further breast segmentation (supplementary S1). The %Dense was calculated as below:

(1) $$\%\mathrm{Dense}=\frac{\mathrm{dense}\;\mathrm{area}}{\mathrm{total}\;\mathrm{breast}\;\mathrm{area}}\times 100\%$$

### 2.3. Body Composition

Body composition variables were measured by trained research assistants on the same morning session with female wearing light clothing after at least 8 hours of fasting. Body height and weight were measured using a digital scale and the body mass index (BMI) was calculated as body weight (kg) divided by squared height (m^2^). Waist circumference (WC) was measured at the midpoint between the lowest rib and the iliac crest. Hip circumference (HC) was measured at the maximum protrusion of the gluteal region. A multi-frequency (5, 50, and 250 kHz) bioelectrical impedance analyzer (IOI-353; Jawon Medical, Kungsan, South Korea) using the tetra-polar electrode method was used to measure the body fat percentage (%BF). After the gender, age, and height were entered into the device, women were asked to stand in a stable position with bare feet. Bioelectrical impedance analysis was not performed in subjects with pacemakers or metal implants.

## 3. Statistical Analysis

SPSS (version 22.0; IBM Corp., Armonk, NY, USA) was used for data analysis and graph generation. Missing values on WC, HC, and %BF were imputed using BMI as a predictor in the regression imputation model. Quadratic weighted kappa coefficient was calculated to measure the intra-observer variability for both assessments of breast density [28]. Age-related differences were analyzed by dividing subjects into groups at 5-year intervals. One-way ANOVA was used to access the differences between groups. Linearity and deviation from linearity were also tested to assess the data for homoscedasticity. The Pearson correlation coefficient was used to measure the linear correlation between two variables. Partial correlation was conducted to control for the effects of potential confounders. The receiver operating characteristic (ROC) with area under the curve (AUC) was used to illustrate the diagnostic performance of the tested variable in discriminating between dense and fatty breasts. The statistical significance level was set at *p* = 0.05.

## 4. Results

Of the 2311 mammography records collected, 143 with BI-RADS 0 of 3–6 were excluded, leaving 2168 mammograms from 2035 women for the final statistical analysis. The average age at screening was 51.4 years (range, 16–86 years). The median time between mammography and body composition measurement was 0 days (interquartile range 3 days). Missing data were present in 1.20% (*n* = 26) of the %BF determinations and 0.42% (*n* = 9) of the WC and HC measurements. The concordance between two BI-RADS breast density reports assessed on two occasions 3 months apart by the same radiologist was very good (weighted kappa = 0.907, *p* < 0.001).

Subject characteristics by age are shown in Table 1. The test for linearity was significant (*p* < 0.001, Table 1) for all variables, suggesting a linear relationship between age and all tested body and breast composition variables. The test for deviation from linearity was also significant (*p* < 0.05, Table 1) for WC, HC, total breast area, and breast fat area, suggesting a nonlinear relationship in addition to the linear component across age groups for these variables. In contrast, increased age was also associated with a consistent linear decline in %Dense and dense area whereas a linear increase in BMI and %BF (*p* > 0.05 by test of deviation from linearity, Table 1). The mean total, dense and non-dense breast areas were 92.6, 22.8, and 69.8 cm^2^, respectively. The mean %Dense was 25.8%.

The distribution of breast density by age is shown in Table 2.

**Figure 1:**
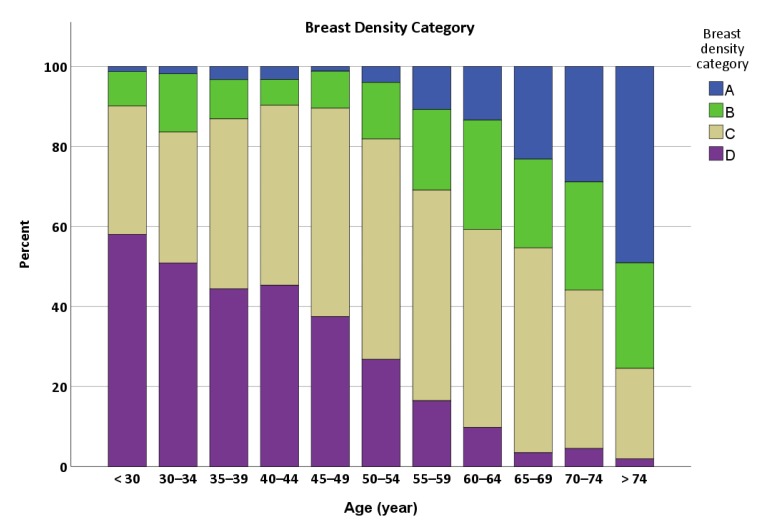
Histogram showing the proportion of breast density categories by age group, using the 5th edition of the Breast Imaging Reporting and Data System (BI-RADS). Note: Category A, almost entirely fatty; category B, scattered areas of fibroglandular density; category C: heterogeneously dense; and category D: extremely dense.

A total of 405 women (18.7%) were younger than 40 years old. For all age groups, 206 women (9.5%) had category A density on mammography, 349 (16.1%) had category B, 1020 (47.0%) had category C and 593 (27.4%) had category D. The frequency distribution of breast density categories across age groups (Figure 1) shows a decline in the proportion of extremely dense breast with increasing age, accompanied by an increase in the proportion of fatty breast.

The proportion of extremely dense breast was 58.0% in women < 30 years, declined until age 35, remained about the same for those 35–44 years, then sharply declined to just 1.9% in women > 74 years. By contrast, the proportion of almost fatty breast was only ≤ 4% in women < 55 years, followed by a rapid increase to 49.1% in women > 74 years. Notably, at least 80% of mammograms in women < 55 years were classified into extremely dense or heterogeneously dense breast, while the proportion of dense breasts in mammograms was still 59.3% in women aged 60–64 years. For women aged < 45, 45–74 and > 74 years, the predominant breast density category for each was extremely dense, heterogeneously dense and almost fatty, respectively (Table 2).

**Figure 2:**
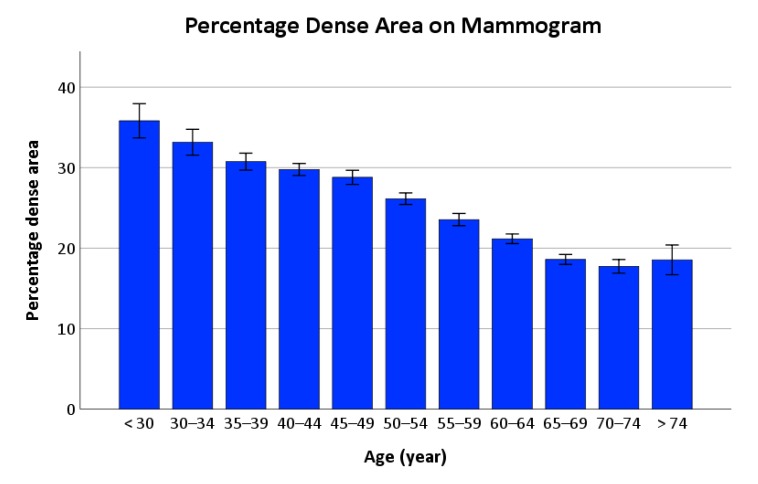
Bar chart showing percentage dense area of the breast for various age groups. Note: The error bars depicted the standard error of the mean. The test for linearity showed a significant difference between groups (*p* < 0.001) but the deviation from linearity was non-significant (*p* = 0.514).

The effect of age on %Dense is shown in Figure 2. The mean %Dense declined from 35.8% in women < 30 years to 18.5% in women > 74 years (Figure 2). The test for linearity was significant (*p* < 0.001) but that for deviation from linearity was non-significant (*p* = 0.514), indicating an age-related decline in %Dense, the rate of which was consistent across age. Figure 2 showed that changes in %Dense occur with age, but these changes may be affected by body composition.

Table 3 shows that all test variables were significantly correlated (*p* < 0.001), but with different degree of strength (for different values of *r*). A strong to very strong correlation was seen between all pairs of body composition variables (*r* = 0.602–0.816), while body composition variables had a weak correlation with %Dense (*r* = −0.326–(−0.324)) and a weak to moderate correlation with age (*r* = 0.223–0.424), except for a very weak correlation between HC and age (*r* = 0.080). A partial correlation was then calculated to test the correlation between %Dense and age after eliminating the influence of body composition variables (Table 3). The correlation remained significant (*p* < 0.001). While still significant, the partial correlation was less than the simple correlation, implying that the relationship between %Dense and age was in part due to body composition.

**Figure 3:**
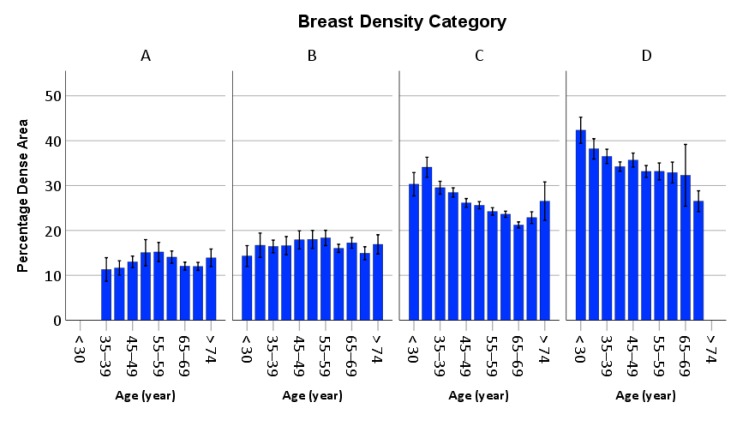
Mean percentage dense area of the breast according to age for the four breast density groups, measured according to using the 5th edition of the Breast Imaging Reporting and Data System (BI-RADS). Note: The error bars depicted the standard error of the mean. Category A, almost entirely fatty; category B, scattered areas of fibroglandular density; category C: heterogeneously dense; and category D: extremely dense.

The means of %Dense in BI-RADS categories A, B, C, and D were 13.2%, 16.9%, 25.8%, and 35.5%, respectively (*p* < 0.001 by ANOVA). Subgroup analyses were also performed to compare the effect of age on %Dense in each breast density category (Figure 3).

Subgroups with < 3 subjects were excluded from analysis. The mean %Dense was significantly associated with age for categories C and D (*p* ≤ 0.001), but not for categories A and B (*p* > 0.05). After controlling for body composition variables, increase in %Dense was still significantly correlated with increasing age for categories C and D (*p* ≤ 0.001).

**Figure 4:**
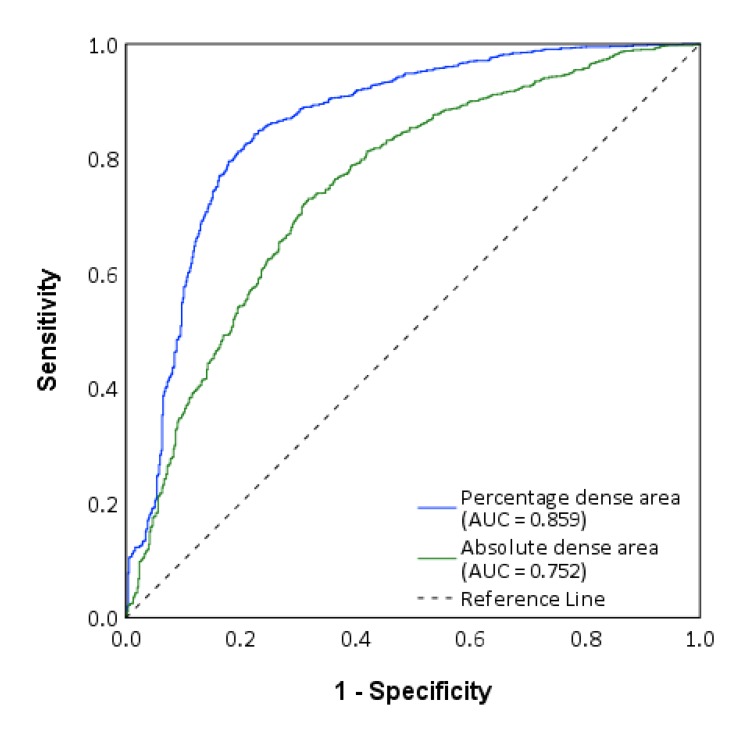
The receiver operating characteristic (ROC) curve for sensitivity and specificity of determining the absolute dense area and percentage dense area in discriminating dense from fatty breasts. AUC, area under the ROC curve.

In the 5th BI-RADS lexicon, breast composition is classified according to the masking effect and pattern of the dense tissue, not the amount of dense breast area. Therefore, neither the absolute nor the relative dense breast area is guaranteed to agree with the visual assessment scale of BI-RADS. In this study, the absolute dense breast area had an acceptable discriminatory ability to distinguish dense breasts from fatty breasts (AUC = 0.752, Figure 4), while the relative dense area (%Dense) had a good discriminatory ability to distinguish dense breasts from fatty breasts (AUC = 0.859, Figure 4). The overall discrimination ability to differentiate dense and fatty breast by the 5th BI-RADS was higher for %Dense compared to that for absolute dense breast area. The optimal cut-off value of %Dense on the ROC curve was 18.4%, with an optimal sensitivity of 77.7% and a specificity of 84.3% (Figure 4).

## 5. Discussion

Breast cancer is the most common cancer in women aged 30–59 years worldwide [29], and therefore, young women may be motivated to engage in early mammography screening. However, the benefits of screening mammography in women < 40 years have not been established and exposure to ionizing radiation involves some risk [30]. Another disadvantage of early mammography is that young women tend to have dense breasts and hence less effective mammography. Therefore, more scientific evidence in addition to economic analysis is needed to guide the screening mammography recommendations for younger women at the population and individual levels. It is well established that mammographic density is negatively correlated with age in women > 40 years old, regardless of ethnicity [23,24,31]. Unfortunately, little is known about the breast density distribution in women < 40 years because most screening mammography guidelines recommend a starting age of 40–50 years. To fill in this gap in knowledge, this study presented qualitative and quantitative breast density changes across a wide range of ages. In this community-based screening, more than 80% of mammograms were classified as dense breasts (BI-RADS categories C or D) in Taiwanese women aged from < 30 years to 50–54 years. Notably, the percentage of dense breasts in Taiwanese women aged 60–64 years in our study was comparable to those in Western women aged 40–44 years [32]. These results imply a similar sensitive of screening mammography for Asian women in their 60s and Western women in their 40s.

Mammographic breast density can be classified by morphology and quantity of dense tissues. The association between breast density and breast cancer was first described by Wolfe [33,34], who graded four breast parenchymal patterns on mammography, as follows: N1; P1; P2; and DY. The cancer rate in the DY group was 37 times greater than that in the N1 group. Since the publication of the Wolfe classification in 1978, several mammographic density classifications have been published. The Boyd classification, published in 1982 [35,36], is a six-grade categorization based on the percentage of dense breast area (A, 0%; B, 0–10%; C, 10–25%; D, 25–50%; E, 50–75%; F, > 75%). A modification of the Wolfe classification was published by Tabár [37] in 1997, which divided breast density into five categories according to the breast parenchymal pattern. The BI-RADS lexicon is the most common reporting system, which provides standardized reporting, assessment, and classification for breast imaging. The selection of descriptors in the BI-RADS lexicon is based on the ability to discriminate benign from malignant breast disease. Breast composition was not recognized as part of the reporting guidelines for mammography until the announcement of the 4th edition BI-RADS in 2003. In the 4th BI-RADS edition, breast parenchymal density was allocated into four categories based on the percent of fibroglandular tissue within the breast (< 25%, 25–50%, 51–75%, or > 75%) [38]. In the current 5th edition BI-RADS published in 2013, this percentage-based reporting system was removed and visual inspection of breast density instead of counting the proportion of dense breast was recommended [39].

In general, the classifications can be divided into quantitative and qualitative assessments. The Boyd classification and 4th BI-RADS edition are primarily quantitative methods, whereas the Wolfe classification, Tabár classification, and 5th edition BI-RADS are qualitative. Despite the similarities in the emphasis on the potential masking effect of dense breast tissue, discrepancies may exist between these classifications. In our study, the age-related variation in breast composition was explored quantitatively and qualitatively. In agreement with other studies [23,24,32], we observed an increasing proportion of fatty breasts and a decreasing proportion of dense breasts with increasing age. An age-dependent decline in the percentage of dense breast area was also found in our study. As expected, both quantitative and qualitative methods identified age effects in breast density.

Interestingly, breasts in categories C and D showed an age-related decline in the percentage of dense breast area but not those in categories A and B. The reasons for discrepancy by breast density category in the decline in age-related percentage of dense breast is not clear. The difference between the purely visual assessment and computer-assisted quantification in this study could not totally explain the results. Aging is associated with regression in breast parenchyma, and therefore, younger women are more likely than older women to have almost all dense tissue in their breasts. This difference could explain the relatively higher percentage of dense tissue for young women with category D but might not explain the findings for women with breast density category C. Parameters related to breast compression and image post-processing techniques may also explain these differences. Younger women in our study had generally smaller breasts than older women, and the smaller breast contact area may result in an overall higher compressed breast thickness and hence a generally greater breast density [30]. In quantitative mammographic density analysis, breast segmentation is based on a threshold-based algorithm and a less compressed mammogram may produce more image pixels higher than the threshold. Further investigation is needed to understand the discrepancies between these two assessment methods.

Only two prior studies have reported an age-related distribution of breast density in women <40 years of age [23,24]. Unfortunately, these two studies did not use the current reporting system. Checka et al. [23] reported 249 women < 40 years of age who underwent screening mammography according to the 4th edition of BI-RADS, which showed a proportion of breast density in categories 1, 2, 3, and 4 to be 1%, 18%, 51%, and 30%, respectively [23]. In their study, most of the women were white and had medical insurance. In Korea, Youn et al. [24] reported the breast density distribution in 1484 women aged 30–39 years who underwent screening mammography; the proportion of dense breasts was 94.3%. In the study by Youn et al. [24], mammographic density was analyzed using imaging software and qualitative classification was not available.

Screening mammography aims to detect early signs of breast cancer, such as small lesions and microcalcifications. Therefore, high spatial resolution and high tissue contrast are essential for optimal lesion detection. Mammography is an imaging modality using X-rays, and thus a lesion can only appear in the absence of the silhouette sign on X-ray films. The ability of a mammogram to detect breast cancer depends on differences in X-ray attenuation properties (or tissue contrast) between breast cancer and the surrounding breast tissue. Johns and Yaffe [40] investigated the attenuation coefficient for cancerous fibroglandular and adipose tissue in the breast, finding a wide X-ray attenuation difference between fat and the other tissue types, whereas a very small attenuation difference between breast cancer and fibroglandular tissue, even at low X-ray energies. In addition, microcalcifications are tiny and clustered structures ranging 0.1-1 mm and are best visualized using mammograms with high resolution [41]. Even with the evolution of breast imaging technologies, imaging breast cancer and microcalcifications in a dense breast remains a challenge [2]. Although it is not clear whether the quality or the quantity of dense breast tissue is more important in the prediction of breast cancer risk, it is generally agreed that even small areas of dense breast tissue may mask a nearby breast malignancy. Our study showed a high dense breast rate (≥ 80%) in women < 55 years, which may indicate high recall rates and low sensitivity in these age groups.

The current study had the advantage of recruiting patients who underwent screening mammography, but not diagnostic mammography, and collecting data from a wide range of women, including women <40 years of age in a homogeneous study population. There were several limitations to this study. First, this was a cross-sectional study and does not reflect changes in the same population over time. Second, this study did not consider the effect of menopause on breast density; however, the age of menopause is related to ethnicity and country of residence. In Asian countries, including Taiwan, the mean age is 47–50 years [42,43]. Third, only the right craniocaudal view of the mammogram was used for breast segmentation. Fourth, our study did not use automated segmentation software, which may not work reliably across databases; however, this study used free and open-sourced software with a plug-in function, which is more accessible and practical than commercial software.

## 6. Conclusions

This work characterized the age-specific breast density of Taiwanese women with ages of 16–86 years, using the current 5th edition BI-RADS and quantitative measures. To our knowledge, it fills in the gap of age-specific breast density distribution in women < 40 years. This study also describes the relationship between qualitative and quantitative breast density measures, and age-related changes in breast composition.

## Figures and Tables

**Table 1:** Subject characteristics by age groups.

Age (Years)	No	BMI (kg/m^2^)	PBF (%)	WC (cm)	HC (cm)	%Dense (%)	Breast (cm^2^)	Dense (cm^2^)	Fat (cm^2^)
<30	81	22.8	24.6	73.1	93.8	35.8	86.3	28.8	57.5
		(4.0)	(5.6)	(10.5)	(8.9)	(19.1)	(42.3)	17.3)	(39.2)
30–34	110	23.9	26.0	77.1	95.3	33.2	96.9	29.7	67.2
		(5.2)	(6.0)	(11.6)	(10.0)	(16.9)	(52.4)	17.7)	(49.4)
35–39	214	23.6	26.3	75.9	94.1	30.7	85.2	25.3	59.9
		(4.1)	(5.5)	(10.1)	(8.6)	(15.4)	(33.1)	15.4)	(30.6)
40–44	311	24.2	27.5	77.2	95.1	29.8	85.4	24.8	60.6
		(3.7)	(5.0)	(9.3)	(7.4)	(13.2)	(31.4)	13.3)	(27.0)
45–49	259	24.9	28.7	79.6	96.8	28.8	91.3	25.5	65.8
		(4.0)	(4.9)	(9.6)	(7.8)	(14.1)	(32.6)	15.8)	(29.5)
50–54	276	24.7	29.3	78.8	95.1	26.1	84.1	21.3	62.8
		(3.7)	(5.0)	(8.9)	(7.1)	(11.9)	(29.5)	11.8)	(27.0)
55–59	243	25.1	30.6	80.3	96.1	23.5	93.2	21.1	72.1
		(3.9)	(5.2)	(9.6)	(7.9)	(11.8)	(31.3)	11.8)	(29.0)
60–64	307	26.2	32.1	83.4	96.8	21.2	100.5	20.8	79.7
		(3.8)	(5.2)	(9.2)	(7.4)	(10.5)	(35.4)	12.3)	(32.4)
65–69	203	25.7	32.5	82.2	95.7	18.6	101.3	18.3	83.0
		(3.3)	(4.3)	(8.9)	(7.7)	(8.8)	(29.5)	9.8)	(27.6)
70–74	111	26.3	32.6	84.2	96.8	17.7	104.5	18.0	86.5
		(3.6)	(5.4)	(8.7)	(7.5)	(8.9)	(33.0)	10.7)	(29.9)
>74	53	26.0	33.1	83.9	96.1	18.5	109.5	20.0	89.5
		(4.1)	(4.9)	(10.3)	(8.2)	(13.4)	(33.7)	15.6)	(31.6)
Total	2168	24.9	29.4	79.6	95.7	25.8	92.6	22.8	69.8
		(4.0)	(5.7)	(9.9)	(7.9)	(13.8)	(34.6)	13.8)	(32.3)
Between group statistics									
ANOVA		0.000	0.000	0.000	0.001	0.000	0.000	0.000	0.000
Linearity		0.000	0.000	0.000	0.000	0.000	0.000	0.000	0.000
Deviation from linearity		0.076	0.187	0.009	0.043	0.514	0.000	0.083	0.000

Note: Data were presented with mean and standard deviation (in parentheses). BMI, body mass index; Dense, high density area in mammography; Fat, radiolucent area in mammography; HC, hip circumference; PBF, percentage body fat; WC, waist circumference; %Dense, percentage of high-density areas in mammography.

**Table 2:** Distribution by age group of mammographic density according to the 5th edition Breast Imaging Reporting and Data System (BI-RADS) classification.

Age (Years)	Subjects in BI-RADS Density Categories				
	A	B	C	D	Total (%)
<30	1	7	26	47	81
	1.2%	8.6%	32.1%	58.0%	100.0%
30–34	2	16	36	56	110
	1.8%	14.5%	32.7%	50.9%	100.0%
35–39	7	21	91	95	214
	3.3%	9.8%	42.5%	44.4%	100.0%
40–44	10	20	140	141	311
	3.2%	6.4%	45.0%	45.3%	100.0%
45–49	3	24	135	97	259
	1.2%	9.3%	52.1%	37.5%	100.0%
50–54	11	39	152	74	276
	4.0%	14.1%	55.1%	26.8%	100.0%
55–59	26	49	128	40	243
	10.7%	20.2%	52.7%	16.5%	100.0%
60–64	41	84	152	30	307
	13.4%	27.4%	49.5%	9.8%	100.0%
65–69	47	45	104	7	203
	23.2%	22.2%	51.2%	3.4%	100.0%
70–74	32	30	44	5	111
	28.8%	27.0%	39.6%	4.5%	100.0%
>74	26	14	12	1	53
	49.1%	26.4%	22.6%	1.9%	100.0%
Total	206	349	1020	593	2168
	9.5%	16.1%	47.0%	27.4%	100.0%

Note: Category A, almost entirely fatty; category B, scattered areas of fibroglandular density; category C: heterogeneously dense; and category D: extremely dense.

**Table 3:** Correlation coefficients and partial correlation coefficients between breast density, age, and physical characteristics.

Control Variables			%Dense	Age	WC	HC	BMI	PBF
**None**	%Dense	Correlation	1.000	−0.361	−0.324	−0.226	−0.302	−0.318
		Significance (2-tailed)		0.000	0.000	0.000	0.000	0.000
		df	0	2166	2166	2166	2166	2166
	Age	Correlation	−0.361	1.000	0.276	0.080	0.223	0.424
		Significance (2-tailed)	−0.000		0.000	0.000	0.000	0.000
		df	2166	0	2166	2166	2166	2166
	WC	Correlation	−0.324	0.276	1.000	0.781	0.816	0.699
		Significance (2-tailed)	0.000	0.000		0.000	0.000	0.000
		df	2166	2166	0	2166	2166	2166
	HC	Correlation	−0.226	0.080	0.781	1.000	0.786	0.602
		Significance (2-tailed)	0.000	0.000	0.000		0.000	0.000
		df	2166	2166	2166	0	2166	2166
	BMI	Correlation	−0.302	0.223	0.816	0.786	1.000	0.738
		Significance (2-tailed)	0.000	0.000	0.000	0.000		0.000
		df	2166	2166	2166	2166	0	2166
	PBF	Correlation	−0.318	0.424	0.699	0.602	0.738	1.000
		Significance (2-tailed)	0.000	0.000	0.000	0.000	0.000	
		df	2166	2166	2166	2166	2166	0
WC & HC & BMI & PBF	%Dense	Correlation	1.000	−0.268				
		Significance (2-tailed)		0.000				
		df	0	2162				
	Age	Correlation	−0.268	1.000				
		Significance (2-tailed)	0.000					
		df	2162	0				

Note: For the partial correlation between percentage dense breast (%Dense) and age, excluded were the influence of waist circumference (WC), hip circumference (HC), body mass index (BMI) and percentage body fat (PBF).

## Supplementary Materials

The following are available online at https://www.mdpi.com/1660-4601/17/9/3186/s1, S1: Breast segmentationIllustration of mammogram image segmentation process.
