# Supplementary material for: Age-Specific Breast Density Changes in Taiwanese Women: A Cross-Sectional Study
Source: Int J Environ Res Public Health. 2020 May 4;17(9):3186. doi: 10.3390/ijerph17093186 (PMC7246480; doi:10.3390/ijerph17093186)
Supplement: Supplementary file 1 [file ijerph-17-03186-s001.pdf]

Supporting Material

S1. Breast segmentation.

Percentage mammographic density (%Dense) was defined as the percentage of the breast that is occupied by radiopaque fibroglandular tissue using an open sources and free software (Fiji, https://imagej.net/Fiji) [1]. For each female, the DICOM format of the right craniocaudal mammogram was imported into the Fiji software for further breast segmentation. The procedure of breast segmentation was illustrated in Supplementary Figure 1. Pre-processing was started by manually cropping non-breast portions of the selected image (e.g., chest wall, view tags, and artifacts). Contrast enhancement using the histogram equalization technique was then performed to better identify the breast boundary. Then, an auto-threshold was applied to separate the breast contour from the background and the area of the total breast was measured. The bright intensity in the air-skin interface had similar intensity to fibroglandular tissue and may thus cause artifacts during the thresholding process. Therefore, the air-skin interface was removed prior to further segmentation. In this study, an erode function was used to remove the pixels from the nearest 50–100 pixels from the edge of the breast (radius set to 50–100). Segmentation of the dense breast area was performed using auto-thresholding and the dense area was extracted.

The %Dense was calculated as below:

$$\%\text{Dense} = \frac{\text{dense area}}{\text{total breast area}} \times 100\%$$

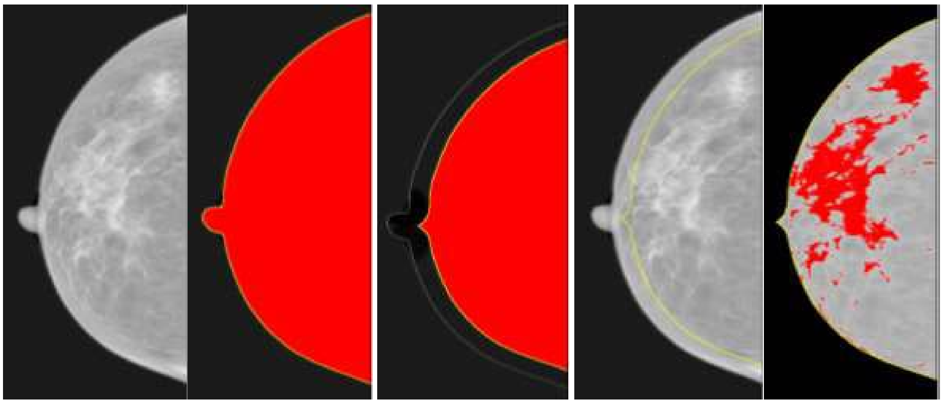

Figure 1. Illustration of mammogram image segmentation process.

Note: (A) Original image, (B) breast contour delineation, (C) removal of skin edge, (D) select area for segmentation, and (E) dense breast thresholding.

References

1. Schindelin, J.; Arganda-Carreras, I.; Frise, E.; Kaynig, V.; Longair, M.; Pietzsch, T.; Preibisch, S.; Rueden, C.; Saalfeld, S.; Schmid, B., et al. Fiji: An open-source platform for biological-image analysis. *Nat. Methods* **2012**, *9*, 676–682.
